# Prescription database analyses indicates that the asthma medicine montelukast might protect against dementia: a hypothesis to be verified

**DOI:** 10.1186/s12979-017-0102-7

**Published:** 2017-08-31

**Authors:** Bjørn Grinde, Bo Engdahl

**Affiliations:** Department of Aging, Norwegian Institute of Public Health, Box 4404 Nydalen, 0403 Oslo, PO Norway

**Keywords:** Montelukast, Prescription database, Dementia, Cognitive decline, Leukotriene

## Abstract

**Background:**

It has recently been shown that the leukotriene receptor antagonist montelukast rejuvenates aged brains in rats. The question is whether this commonly used, systemic, anti-asthmatic medicine has a similar effect in humans?

**Results:**

We approached this issue by doing statistical analyses based on the Norwegian Prescription Database. The Database lists all prescription-based medications in Norway, but not drugs given to people who are in hospitals or nursing homes. The question asked was whether users of montelukast, compared to users of inhalation asthma medicine, live longer, and are less likely to develop dementia. A small, non-significant protective effect on the use of dementia medicine became significant when adjusting for other prescriptions (based on the notion that montelukast users on average are less healthy). A possible protective effect was substantiated by looking at the lack of prescriptions as a proxy for dementia-related residency in nursing homes, and the risk of death.

**Conclusions:**

The present results suggest that montelukast may alleviate the cognitive decline associated with human aging. However, further data, preferably based on controlled clinical trials, are required.

## Background

According to Marschallinger et al. [1], six weeks of treatment with the asthma medicine montelukast rejuvenates the brain of aging, but otherwise healthy, rats (20 months old, considered equivalent to 60 years in humans). The observation offers hope for finding a remedy that can reduce human, age-related cognitive decline. Montelukast inhibits inflammatory processes by acting as a leukotriene receptor antagonist. There are considerable data suggesting that inflammation plays a role in age-associated disorders of both body and brain [1–4].

The question of whether montelukast has a positive effect on humans is difficult to resolve, as it is ethically questionable to set up clinical trials that involve the use of prescription medicine on healthy individuals. To circumvent this problem, we analysed data from the Norwegian Prescription Database (NorPD) http://www.norpd.no/. A clinical trial should be more acceptable if there is sufficient data suggesting a protective effect. NorPD registers all prescriptions in Norway, but does not cover medication given to people in hospitals or nursing homes. The database allows for asking questions such as whether subjects given montelukast are later less likely to receive medication related to dementia, when compared with individuals using inhalation asthma medicine.

In Norway, with a population of about 5 million, there were 37,445 registered users of montelukast in 2014, of which approximately half were 50 years or older. The use dates back to the nineties. The primary indication (in adults) is the additional treatment of asthma in cases where inhalation medications are insufficient to control the condition. Previous data suggest that asthma patients have an increased risk of both dementia [5, 6] and death [7], which is why we used subjects receiving inhalation medication as a control group. It is, however, expected that users of montelukast have a more severe form of asthma; a conjecture that was supported by the present data. In separate analyses, it was therefore corrected for the use of additional medication as a proxy for general health.

One problem when looking at the use of dementia medicine in Norway is that these drugs are in principle prescribed to people with Alzheimer’s disease (AD). It is, however, likely that some of the patients offered medication have other forms of dementia. With this in mind, we considered it useful to compare with patients taking medication associated with Parkinson’s disease (PD). Approximately half of those diagnosed with PD will develop dementia [8, 9], but this diagnosis is less likely to be confused with other forms of dementia. Both AD and PD are likely to reflect aetiologies somewhat different from the normal cognitive decline associated with aging [10], aetiologies that may or may not involve the leukotriene signalling pathway. Based on the above discussion, one might expect a limited protective effect of montelukast on the use of dementia medicine, but perhaps not on Parkinson’s medication.

NorPD offers two alternative options for probing the effect of montelukast. Individuals who stop receiving prescriptions for an extended period, while still being alive, are likely to be admitted to an institution. Long-term stay in institutions for elderly people generally means nursing homes, and dementia is the most common reason why people are committed to nursing homes in Norway (the prevalence of dementia in nursing homes residents has been estimated to be approximately 80% [11]). We consequently used the lack of prescriptions for a period of at least one year as a proxy for cognitive decline. This group would include all forms of dementia, of which perhaps half suffers from other forms than those related to AD or PD [8, 12]. A final option was to consider whether montelukast protects against death.

## Results

The subset of NorPD data used in the present analyses included all prescriptions given to people offered asthma-related medicine who were 60 years or older in 2014. The current version of the database includes the period from 2004 to 2015. Drugs are defined according to the Anatomical Therapeutic Chemical (ATC) classification system, and can be listed as the number of Defined Daily Doses (DDDs) dispensed. The study cohort was further limited to individuals (203,473) who had at least two prescriptions of montelukast (ATC code R03DC03) or inhalation type asthma medication (codes R03AK or R03BA – that is, primarily corticosteroids) during the period covered in the database (Table 1). The mean age of the study cohort was 75.2 years in 2014.

**Table 1:** Characteristics of the study cohort (elderly on asthma medicine)

Study group	Total	Mean age (SD)^a^	Males (%)	Montelukast^b^ (%)	Total drugs in DDDs (SD)^c^
All subjects	203,473	75.2 (9.7)	89,755 (44.1)	23,636 (11.6)	5.1 (4.6)
Montelukast	23,636	73.1 (9.0)	8964 (37.9)	-	6.3 (4.3)
Dementia medicine	6453	83.6 (7.7)	2502 (38.8)	602 (9.3)	5.6 (3.2)
Nursing home^d^	5970	77.8 (10.4)	2463 (41.3)	507 (8.5)	4.2 (3.5)
Death	61,434	82.7 (9.5)	31,152 (50.7)	5514 (9.0)	7.1 (6.3)
Parkinson’s medicine	7140	76.1 (9.3)	2849 (39.9)	955 (13.4)	6.3 (3.9)
Diabetes medicine	23,747	74.8 (9.1)	11,920 (50.2)	2887 (12.2)	7.8 (4.3)

^a^Age in 2014

^b^At least two prescriptions

^c^Mean Defined Daily Doses (DDDs) prescribed of any drug

^d^It was assumed that a majority of those who did not receive any prescription for more than a year were admitted to a nursing home. Living in a nursing home can be used as a proxy for cognitive decline in that some 80% of Norwegian nursing home residents have dementia [11]

The follow-up groups included: 1) users of *montelukast* (11.6%); 2) users of *dementia medicine* (code N06D; 3.2%); 3) those who presumably had been admitted to a *nursing home* (2.9%); 4) those who *died* (30.2%); 5) users of *Parkinson’s medicine* (code N04; 3.5%); and 6) users of type 2 *diabetes medicine* (codes A10BA, A10BB, and A10BH; 11.7%). Based on total drug consumption, subjects belonging to most follow-up groups had more health problems compared to the overall average (Table 1). Similar results were found when looking at prescription of cardiovascular medicine (codes B01A, C07, and C09; data not shown). In separate analyses, it was consequently adjusted for a combination of total DDDs, cardiovascular medications, and sex (as women tended to be overrepresented).

The prevalence of montelukast use was lower in the following three groups: dementia, nursing home, and death – compared to the complete study group – while slightly higher in the PD and diabetes groups (Tables 1 and 2). Although these figures are indicative, further analyses are required in order to propose a protective effect.

**Table 2:** Association between montelukast use and risk of different out-comes (significantly protective hazard ratios in bold)^a^

Study group	Cases/Total (%)		Hazard ratios (95% CI)		
	Montelukast pos	Montelukast neg	Age^b^	Unadjusted	Adjusted
Dementia medicine^c^	489/23,521 (2.1)	4967/178,884 (2.9)	>60	0.94 (0.85–1.03)	**0.89** (0.81–0.98)
Nursing home^d^	507/23,634 (2.2)	5459/179,768 (3.1)	60–75	**0.65** (0.57–0.74)	**0.67** (0.59–0.77)
			>75	1.00 (0.88–1.13)	0.99 (0.87–1.13)
Death^d^	5512/23,634 (30.4)	55,857/179,768 (45.1)	60–75	**0.86** (0.82–0.89)	**0.64** (0.61–0.67)
			>75	**0.95** (0.92–0.98)	**0.81** (0.78–0.84)
Parkinson’s medicine^e^	691/23,371 (3.0)	4550/178,134 (2.6)	>60	1.21 (1.12–1.32)	1.06 (0.98–1.15)
Diabetes medicine^e^	1523/22,270 (7.3)	11,247/170,157 (7.1)	>60	1.01 (0.96–1.07)	**0.85** (0.80–0.90)

^a^The hazard ratios are based on comparing subjects using montelukast with those using only inhaling corticosteroids. The adjusted ratios are adjusted for sex, receiving drugs for heart conditions, and having a high consumption of drugs during the follow up period. The discrepancies between the sum of users in each analysis, and the grand total of 203,473 in Table 1, are due to truncations. The total number of person-years follow-up was between 1,346,308 and 1,480,476 (average 7.0–7.3 years per subject)

^b^As of 2014

^c^The adjusted analysis was stratified on having a high consumption of drugs dispensed per day, because this covariate did not meet the proportional hazards assumption

^d^As the effect of montelukast did not meet the proportional hazards assumption, the model was fitted by splitting into two age-periods, 60–75 years and >75 years

^e^The adjusted analysis was stratified on having a high consumption of drugs dispensed per day, and prescription of cardiovascular medicine, because these covariates did not meet the proportional hazards assumption

The calculation of hazard ratios (HRs), based on Cox regression analysis, suggested a slight, but not significant, tendency toward a protective effect of montelukast on the later use of dementia medication (Table 2, unadjusted). The effect did reach significance when adjusting for the above mentioned factors. However, as pointed out in the Introduction, the use of dementia medicine is not a good indicator of general, age-related cognitive decline. We therefore analysed a lack of prescriptions for at least a year as a proxy for subjects being committed to nursing homes. Nursing home residency implies a high risk for any form of dementia [11]. In this case, the data had to be split between those below and above 75 years for analytical reasons. There was a highly significant protective effect on subjects 60–75 years (HR 0.65 and 0.67 for unadjusted and adjusted), while no effect on those above 75. A protective effect was further corroborated by analysing for risk of death during the study period. Again, the data had to be split in two age groups, but in this case, both groups displayed a significant effect of montelukast. Subjects for whom the first prescription of montelukast or inhaling corticosteroids happened after the follow-up events (e.g., the use of dementia or PD medicine), were removed from the regression analyses (truncated).

As these analyses only provide correlates, and may be biased by unknown confounders, we decided to see how two other types of medication would perform in similar analyses. PD and type 2 diabetes presumably have aetiologies where leukotrienes have limited impact. We found a slightly increased prevalence of montelukast use in both groups (Tables 1 and 2). Based on Cox regression analyses, montelukast users had an increased risk of PD, which became not significant in the adjusted model (Table 2). In the case of diabetes, there was no effect in the unadjusted analysis, but an apparent protective effect in the adjusted model.

## Discussion

The present data indicate that the use of montelukast might protect against dementia and extend lives in humans. The study cohort included users of asthma medicine who were 60 years or older in 2014. Based on Cox regression analyses, subjects who received montelukast were less likely later to use dementia drugs (Table 2); although the HR was only significant when adjusting for the consumption of other drugs. The protective effect on the use of dementia medicine was relatively small, but further evidence, discussed below, support the conjecture.

Cognitive impairment, which may lead to dementia, is presumably a natural consequence of normal aging; but dementia may also have more specific causes, such as infections, plaque formation, and stroke. The leukotriene-signalling pathway, which is inhibited by montelukast, is primarily expected to contribute to the general, age-related deterioration; although it has been suggested that leukotrienes may also play a role in more specific forms of dementia such as AD [1, 13, 14]. Dementia medicine is, in principle, given to patients with AD (or, more rarely, PD dementia). There are no drugs intended for other forms of dementia, but the NorPD allows for examining a proxy. The study group used on average more than 5 DDDs, which means that each individual was expected to receive several prescription over the course of a year. Those who did not, while still being alive, were likely to live in an institution. Nursing homes are the main type of institution offering long-term stay for elderly. The prevalence of dementia in Norwegian nursing homes has been estimated to be approximately 80% [11]. The use of montelukast reduced the HR for ending up in the nursing home group (Table 2); that is, for the younger fraction of the study group. In order to satisfy the proportional hazards assumption for the Cox regression, we needed to split the study group in two age fractions. A similar age-effect was observed with the dementia group (data not shown).

The above data are compatible with the following account: Montelukast has none, or limited, protective effect against AD, but may help prevent other forms of dementia, and a minor fraction of these is given dementia drugs. A larger fraction of nursing home residents has a form of dementia that can be prevented or alleviated by montelukast; however, the medicine needs to be taken while the person is still relatively young.

Drugs associated with two other diseases were included as controls. PD, and thus Parkinson’s dementia, is likely to have an aetiology different from that of the previously mentioned conditions. According to the present analyses, montelukast users appeared to have an elevated risk of using PD medicine, but the HR was not significant in the adjusted model. These data suggests that the leukotriene-signalling pathway is not involved as a causal agent. The increased risk may reflect either a protective effect of this pathway, or that montelukast users are less healthy and thus more at risk.

A range of data support the idea that the immune system can impact on the aetiology of perhaps most forms of neurodegenerative disorders (for a broader discussion, see [1–4]), yet the overall effect could be either protective or causal [15, 16]. Inflammation helps remove cellular debris (including plaque-forming proteins). It is conceivable that in the early stages of neurodegeneration, inflammation is destructive – as it also cause tissue damage – while in later stages, with a greater burden of debris, the balance tips in favour of inflammation. Additional information comes from the use of non-steroidal anti-inflammatory drugs (NSAIDs). NSAIDs have not been successful in the treatment of AD [17]; on the other hand, previous use has been associated with a reduced risk of AD and general dementia [18, 19], but not PD [20]. Moreover, a study of genetic polymorphisms for key genes associated with inflammation (cyclooxygenase-2 and 5-lipoxygenase) found that alleles expected to cause increased expression, and thus an increase in inflammatory factors, are overrepresented in AD patients [21]. 5-Lipoxygenase is involved in the generation of leukotrienes. A short-term, randomized controlled trial with NSAIDs did not find any significant protective effect on AD [22]. These observations support the view that early use of drugs designed to reduce inflammatory processes may have a beneficial role, at least in the case of certain forms of dementia. The balance, as to positive or negative effect of inflammation, may depend on stage of neurodegeneration, aetiology, dose/duration of treatment, and the branch of the inflammatory machinery being inhibited. NSAIDs affect the cox/prostaglandine pathway, while montelukast is a leukotriene antagonist.

We also analysed the effect of montelukast on dying. Again, the data had to be split in two age groups. In this case, montelukast had a protective effect on both groups, but a more pronounced effect on the younger fraction. As suggested above, this observation may reflect that the drug is less useful for those at advanced stages of aging; but other factors may also be involved. For example, in the very old, both dying and admission to a nursing home could be due to a larger variety of causes, of which the leukotriene signalling pathway is only involved in some.

A protective effect on dying is in line with the idea that pro-inflammatory processes are associated with a range of age-related disorders, including cardiovascular conditions, and as such act as predictors of mortality [23–26]. In fact, Swedish register data indicate that montelukast reduces the risk of recurrent stroke and myocardial infarction [27]. As to the present data, most follow-up groups had a higher level of DDDs (Table 1), and cardiovascular medication (data not shown), compared to the overall average. One interesting exception was that the montelukast users received slightly less than average heart medication.

Medicine prescribed to people with type 2 diabetes was used as another control. In this case, there was no effect in the unadjusted data, while the adjusted model appeared to indicate a protective effect. Pointing in the opposite direction, there were actually more montelukast users in the diabetes group compared to the total study group. Although inflammation has been proposed as part of the aetiology of type 2 diabetes [23], the observed reduction in HR reflects, perhaps, that these subjects use a particularly high level of other medications (Table 1). The adjusted data may consequently be biased. The diabetes data emphasize the caution required when interpreting correlates obtained from NorPD. The adjustments performed may be more or less pertinent, and there may be confounders that are not adjusted for.

The use of psychotropic drugs may affect brain aging. We consequently investigated whether there was a bias as to the use of the following drugs: N02 – Analgesics, N05 – Psycholeptics, N06A – Antidepressants, N06B – Psychostimulants, and N07 – Other nervous system drugs. The use was slightly more prevalent among the montelukast group, reflecting their elevated, total DDDs (data not shown). If psychotropic drugs increase the likelihood of dementia, the difference would imply that the present results underestimate the positive effect of montelukast.

### Limitations

Besides the obvious problem that the hazard ratios are based on correlations and not causation, we see two main caveats regarding the present study. For one, it employs prescription data as proxies for various conditions, as there is no direct information as to diagnostics in NorPD; and two, the database offers limited options for testing confounders. The main issues regarding these two caveats are discussed below.

The primary indication for prescribing dementia medicine is AD, although one of them (rivastigmine, which stands for some 30% of the prescriptions), can also be used for PD-related dementia. We do not know how often these drugs are prescribed for non-AD dementia. The primary use of PD medication is to alleviate symptoms associated with this disease, but one of the drugs, pramipeksol (code N04 BC05), can also be dispensed for restless legs syndrome. This is a relatively common syndrome; prevalence estimates for our age set range from 5 to 15%, [28], compared to 0.1–1% for PD [29], both increasing with age. However, most likely only a small fraction of restless legs sufferers takes pramipeksol. In 2014, 42% of the subjects on PD medication, received pramipeksol, suggesting that the majority of the PD group has this condition. As to asthma medicine, the code R03 stands for *Drugs for obstructive airway diseases*. The majority presumably has an asthmatic ailment, but the inhalation drugs (not montelukast) may also be prescribed for related conditions such as chronic obstructive pulmonary disease. Montelukast, on the other hand, may be prescribed to treat allergic rhinitis. On a similar note, although lack of prescriptions are likely to be a reasonable proxy for admission to nursing homes, some individuals may end in this group if they have permanently moved abroad, or stop using prescriptions for other reasons. Norway has approximately 40,000 nursing home residents in a population where 700,000 are 65 years or older [30]. Our nursing home group comprised 2.9% of those 60 years or older (Table 1), the numbers are at least compatible with the conjecture that a majority of those included actually are nursing home residents.

The above limitations seem more likely to point in the direction of underestimating, rather than overestimating, a possible effect of montelukast on cognitive decline. The second caveat is perhaps the more problematic. A bias in prescription practises can easily confound the data. For example, the educated and rich may be more likely to seek medical help and obtain medications. Although Norway has a National Insurance Scheme that includes everyone, we would still have preferred to evaluate the effect of socioeconomic factors, but relevant data is not available in NorPD. Moreover, we see no obvious reasons why such a bias should bend the data in any particular direction. Another example is confounding by contraindication. If the prescription of montelukast is contraindicated for some conditions related to the outcomes, the effects may have been inflated. Again, we are not aware of any particular problem pointing in this direction.

## Conclusion

Dementia is arguably the biggest challenge for the future of health-care systems worldwide [31], any measure that can alleviate this burden is highly desirable. The present results do not prove that montelukast will help, placebo-based trials are required to obtain more conclusive evidence. We hope the present data is sufficient to make this option ethically acceptable.

## Methods

### Study cohort

The analyses were based on data from the Norwegian Prescription Database (NorPD; www.norpd.no). The present version of NorPD contains information about all prescribed drugs dispensed at pharmacies to individual patients by any prescriber (within Norway) between January 1, 2004 and December 31, 2015. Medication given patients in nursing homes or hospitals are not recorded in the NorPD, nor are over-the-counter drugs. Available information includes sex, age, the dates on which drugs were dispensed, drug information, and number of Defined Daily Doses (DDDs) [32].

The present study cohort included all who had received two or more prescriptions of either montelukast (ATC code R03DC03) or inhaling corticosteroids (code R03AK or R03BA), and who were 60 years or older in 2014. Two prescriptions, rather than just one, were chosen in order to avoid including subjects who did not actually use the medication. Information on all other prescriptions, and year of death, were also retrieved. Individuals included in the *montelukast group* may have dispensed inhaling corticosteroids, whereas individuals in the *inhaling corticosteroids group* did not have any recorded prescription of montelukast. Drug prescriptions were also used as a proxy for other diseases: Individuals who had received at least one prescription of dementia drugs (code N06D; i.e., memantine, donepezil, rivastigmine, or galantamine) were included in the *dementia group*; Parkinson’s drugs (code N04) in *Parkinson’s group*; and type 2 diabetes drugs (codes A10BA, A10BB, and A10BH) in the *diabetes group*.

### Assessment of potential covariates

The mean number of DDDs dispensed was used as a proxy for drug consumption. The number of DDDs used by an individual was calculated by the following equation:

$$ \mathrm{DDDs}=\frac{\mathrm{number}\  \mathrm{of}\  \mathrm{tablets}\  \mathrm{dispensed}\times \mathrm{amount}\  \mathrm{of}\  \mathrm{drug}\ \mathrm{per}\ \mathrm{tablet}\ \left(\mathrm{mg}\right)}{\mathrm{DDD}\ \mathrm{of}\  \mathrm{the}\  \mathrm{drug}\ \left(\mathrm{mg}\right)} $$

As a gross measure of health, we calculated the mean DDD of all drugs dispensed per day by dividing the sum of DDDs during the study period for each individual by the number of days between the first date of inclusion and the end of the follow-up or when censored. The total drug consumption was categories into four categories: <=5 DDDs/day, 5–10 DDDs/day, 10–15 DDDs/day, and >15 DDDs/day. Drug exposure, assessed as at least one prescription of the following drugs, was used to construct proxies for cardiovascular problems: Antithrombotic agents (code B01A), beta blocking agents (code C07), and agents acting on the renin-angiotensin system (code C09). These proxies were dichotomized into exposed or not and used as covariates in the analyses to adjust for medical conditions that involve higher risk of the different outcomes.

### Statistical analyses

All descriptive analyses and survival analyses were conducted using Stata version 14.0.

We computed the age for each participant at the date exiting the study as either the date of failure (for example the first prescription of an anti-dementia drug), the year of death, or the end of follow-up (2015), whichever came first. The relative risk of the outcomes associated with dispensing montelukast, compared to the reference group who only dispensed inhaling corticosteroids (the hazard ratio), was calculated using Cox regression. Age was treated as survival-time in the analyses. These analyses were performed unadjusted as well as by adjusting for sex, cardiovascular medication, and the mean number of DDDs dispensed of any drugs.

The effect parameters are possible to estimate in Cox regression without any consideration of the underlying hazard function, if the proportional hazards assumption holds. Testing the assumption of proportional hazards was performed by the stphtest in Stata. Analyses were stratified on covariates for which the effect did not meet the proportional hazards assumption. In models in which the assumption did not hold for the exposure variable montelukast, we estimated separate effects for two different age periods, 60–75 years and above 75 years.

All statistical tests were two-tailed and calculated at a 95% confidence interval (*p* < 0.05).

### Censoring and truncation

Subjects were censored at the date of their last prescription. Drugs received by patients in nursing homes or other institutions are not reported to the NorPD. Hence, people in the present data set, for whom the NorPD registrations stopped and who still remained alive for one year or more, were included in the *“nursing home”* group (other institutions, such as hospitals, are unlikely to retain patients for extended periods). Individuals who died before their last prescription were censored at the year of death.

Subjects who dispensed a dementia drug, a Parkinson drug, or a type 2 diabetes drug before the second prescription of montelukast or inhaling corticosteroids were truncated.

## Abbreviations

AD: Alzheimer’s disease
ATC: Anatomical Therapeutic Chemical
DDD: Defined Daily Dose
HR: Hazard ratio
NorPD: Norwegian Prescription Database
NSAID: Non-steroidal anti-inflammatory drugs
PD: Parkinson’s disease
